# Flower transcriptome dynamics during nectary development in pepper (*Capsicum annuum* L.)

**DOI:** 10.1590/1678-4685-GMB-2018-0267

**Published:** 2020-05-29

**Authors:** Ming-hua Deng, Kai Zhao, Jun-heng Lv, Jin-long Huo, Zhu-qing Zhang, Hai-shan Zhu, Xue-xiao Zou, Jin-fen Wen

**Affiliations:** College of Horticulture, Yunnan Agricultural University, Kunming, P. R. China.; Hunan Academy of Agricultural Science, Changsha, P. R. China.; College of Agriculture and Life Sciences, Cornell University, Ithaca, NY, USA.; Faculty of Architecture and City Planning, Kunming University of Science and Technology, Kunming, P. R. China.; Faculty of Animal Science and Technology, Yunnan Agricultural University, Kunming, P. R. China.

**Keywords:** Capsicum annuum L., flower transcriptome, nectar biosynthesis, nectary development, gene

## Abstract

The measurement of gene expression can provide important information about gene function and the molecular basis for developmental processes. We analyzed the transcriptomes at three different developmental stages of pepper flower [sporogenous cell division, stage (B1); pollen mother cell meiosis, stage (B2); and open flower (B3)]. In the cDNA libraries for B1, B2, and B3: 82718, 77061, and 91491 unigenes were assembled, respectively. A total of 34,445 unigene sequences and 128 pathways were annotated by KEGG pathway analysis. Several genes associated with nectar biosynthesis and nectary development were identified, and 8,955, 12,182, and 23,667 DEGs were identified in the B2 vs B1, B3 vs B1, and B3 vs. B2 comparisons. DEGs were involved in various metabolic processes, including flower development, nectar biosynthesis, and nectary development. According to the RNA-seq data, all 13 selected DEGs showed similar expression patterns after q-PCR analysis. Sucrose-phosphatase, galactinol-sucrose galactosyltransferase, and sucrose synthase played very important roles in nectar biosynthesis, and *CRABS CLAW* could potentially be involved in mediating nectary development. A significant number of simple sequence repeat and single nucleotide polymorphism markers were predicted in the *Capsicum annuum* sequences. The new results provide valuable genetic information about flower development in pepper.

## Introduction

Most flowering plants attract pollinators by offering a reward of floral nectar in many plant-pollinator systems. A floral organ called the nectary is responsible for nectar biosynthesis. However, the molecular events associated with the biosynthesis of nectar and nectary development are not clearly understood. To date, only a few individual genes, including *BLADE-ON-PETIOLE* (*BOP*) *1*, *BOP2*, and *CRABS CLAW (CRC)* have been isolated and confirmed to be associated with the development of the nectary (Lee *et al.*, 2005a; McKim *et al.*, 2008). Previous research has shown that *crc* knockout mutant lines failed to develop a nectary, whereas *bop1/bop2* double mutant plants have significantly smaller nectaries along with aberrant morphologies (Bowman and Smyth, 1999; McKim *et al.*, 2008). Furthermore, although *CRC* expression is essential, it cannot promote ectopic nectary development and previous results have indicated that some additional genetic elements might exist that restrict nectary development (Baum *et al.*, 2001). Some other organ identity genes, including *LEAFY*, *AGAMOUS* (*AG*), *SHATTERPROOF1*/*2*, *APETALA2/3* (*AP2/3*), *PISTILLATA* (*PI*), and *SEPALLATA1/2/3* (*SEP1/2/3*), have demonstrated roles in regulating *CRC* expression (Baum *et al.*, 2001; Lee *et al.*, 2005b). However, most of the genes that participate in *de novo* nectar production and development of the nectary have not been identified, which limits our understanding of the pathways and cellular processes critical for nectary development and function.

Pepper (*Capsicum annuum* L.) originated in Central and South America. It is one of the most important economic vegetables in the world and is consumed as a fruit or is processed into other products (Zou, 2002). Nectar is the primary reward offered by plants to attract pollinators. Pepper is a typical allogamy plant and has predominant heterosis. The use of nectaries to attract insect pollination has important potential when attempting to breed peppers using the cytoplasmic male sterile line as the female parent. However, nectar biosynthesis and nectary development in pepper flowers is not clearly understood. Xin *et al.* (2008) reported that the pepper floral nectary is at the base of the ovary and belongs to the ovarial nectary. The nectary is composed of nectariferous tissue and a secretory epidermis, which is covered by cuticle tissue.At the sporogenous cell division stage, the nectary has not yet developed and at the pollen mother cell stage, the nectary is forming, but nectar is not present. Colorless and transparent nectar is produced at the open flower stage.

Pepper nectar contains fructose, glucose, and sucrose. Therefore, the sugar metabolism pathway plays an important role in the biosynthesis of pepper nectar (Rabinowitch *et al.*, 1993; Roldán-Serrano and Guerra-Sanz, 2004; Greco *et al.*, 2011; Pereira *et al.*, 2015).

Next-generation sequencing technologies (e.g*.*, RNA-seq) permit that the whole transcriptome can be sequenced, and they are convenient and rapid methods that can be used to investigate gene expression at the whole-genome level and define putative gene function (Ozsolak and Milos, 2011; Jain, 2012). Over the past few years, many studies have confirmed the efficiency and sensibility of RNA-seq in various biological contexts (González-Ballester *et al.*, 2010; Li *et al.*, 2010; Zenoni *et al.*, 2010; Yang *et al.*, 2011). Rapid progress has been made towards understanding the transcriptional programs associated with the specific development processes of many plant species, but only a few studies have investigated pepper (Ashrafi *et al.*, 2012; Liu *et al.*, 2013; Martínez-López *et al.*, 2014).

To better understand the molecular mechanisms of nectar biosynthesis and nectary development in pepper, the sequencing data from the young pepper flowers obtained from RNA-seq was used to investigate differential gene expressions at three pepper flower development stages. We obtained high-quality reads and assembled unigenes that allowed us to identify the genes involved in pepper flower development, nectar biosynthesis, and nectary development. By comparing the expression patterns of genes at different stages of pepper flower development, we were able to identify several pathways and a large number of differentially expressed genes (DEGs). Some DEGs involved in nectar biosynthesis and nectary development were selected and analyzed by quantitative real-time PCR (qPCR). Our study provides valuable genetic information for the elucidation of pepper flower nectar biosynthesis and nectary development.

## Materials and Methods

### Plant material collection

The pepper cultivar 9704B was grown in experimental fields at the College of Horticulture and Landscape, Yunnan Agricultural University, China. Nectary development was divided into three stages (B1: sporogenous cell division, where the nectary has not yet developed; B2: pollen mother cell, where the nectary is forming, but no nectar is produced; and B3: open flower with nectar production). These stages were based on the cytology results reported by Xin *et al.* (2008). The materials were frozen in liquid nitrogen and then stored at -80 °C until needed.

### RNA extraction and library preparation for transcriptome analysis

Total RNA was isolated using the CTAB reagent method (Invitrogen). RNA from the B1, B2, and B3 stages of flower development was used to construct the sequence libraries. A NanoDrop 1000 spectrophotometer and an Agilent 2100 Bioanalyzer were used to verify RNA quality and quantity prior to further processing. The total RNA was treated with DNase I prior to library construction. Magnetic Oligo(dT) beads were used to purify the poly-(A) mRNA. Adaptor-ligated fragments were generated according to manufacturer's instructions for T4 DNA polymerase, T4 polynucleotide kinase, Klenow 3' to 5' exo-polymerase, and T4 DNA ligase. When the adaptor-ligated fragments were separated on a 1.0% agarose gel, the desired range of cDNA fragments (200 ± 25 bp) were excised from the gel and then the cDNA fragments were enriched and amplified using PCR. The cDNA library was subjected to Solexa sequencing using an Illumina HiSeq2000 sequencing platform (BGI-Shenzhen, Shenzhen, China).

### *De novo* assembly, assessment, and annotation

The raw data processing and functional annotations were performed according to Zhang *et al.* (2015). In brief, the raw sequencing data were transformed by base calling into sequence data, which were stored in fastq format. Adaptor fragments were removed from the raw reads to obtain the clean reads. *De novo* transcriptome assembly of these short reads was performed using the SOAP *de novo* assembling program, which first combined reads with a certain length of overlap to form longer fragments without Ncalled contigs. The reads were mapped back to the contigs. Paired-end reads were used to check the contigs that had come from the same transcript. Secondly, using "N" to represent unknown sequences, SOAP *de novo* connected the contigs to produce the scaffolds. Paired-end reads were then used again to fill in the gaps between the scaffolds. These were designated as unigenes.

For further analysis, we used BLASTX (E-value < 10^-5^) to search the unigene sequences against many protein databases, including the non-redundant database (Nr), SwissProt, the Kyoto Encyclopedia of Genes and Genomes (KEGG), and the Orthologoue Group of proteins (COG) (Natale *et al.*, 2000). Unigene sequences were not used to search the subsequent database(s) if they have hits in one of the named databases. BLAST results then used to extract the coding sequences (CDSs) from the unigene sequences and translate them into peptide sequences. The BLAST results were also used to train ESTScan. If the unigene CDSs had no BLAST hits, then they were predicted by ESTScan, and translated into peptide sequences. Unigene annotation provided information on the expression patterns and functional annotations of the identified genes. For Nr annotation, the BLAST2GO program was used to obtain the GO annotations for the unigenes (Conesa *et al.*, 2005), and WEGO software was used to perform the GO functional classifications for all the unigenes (Ye *et al.*, 2006). The WEGO software was also used to explore the macro-distribution of gene functions for this species.

### Analysis of metabolic pathway genes identified from pepper flowers

The KEGG database and related software applications (http://www.genome.jp/kegg/kegg4.html) were used to analyze the metabolic pathways (Nakao *et al.*, 1999; Kanehisa *et al.*, 2008). The variations that were specific to particular organisms and information about the networks of molecular interactions within cells were obtained from the PATHWAY database. The genes involved in flower development, nectar biosynthesis, and nectary development were selected and analyzed using BLAST annotation of KEGG and the other databases mentioned above.

### Differential gene expression analysis

The KEGG database and related software applications (http://www.genome.jp/kegg/kegg4.html) were used to analyze the metabolic pathways (Nakao *et al.*, 1999; Kanehisa *et al.*, 2008). Transcript expression was calculated by the RPKM method using the following formula: RPKM(A) = 10^6^C / (N × L / 10^3^), where RPKM(A) is the expression of unigene A, C is the number of fragments uniquely aligned to unigene A, N is the total number of fragments uniquely aligned to all the unigenes, and L is the base number in the coding region (CDS) of unigene A. The *p*-value corresponding to the differential transcript expression in two samples was determined according to Audic and Claverie (1997) and the FDR method was used to determine the threshold *p*-values in a number of tests. We used FDR ≤ 0.001 and the absolute value of log2 ratio ≥ 1 as thresholds to consider whether the gene expression difference was significant.

### Gene validation and expression analysis

For the qPCR analysis we selected 13 unigenes that had potential roles in nectar biosynthesis and nectary development for validation. Specific primers for qPCR were designed using Primer Premier 5.0 (Table 1). Total RNA was extracted individually from the B1, B2, and B3 developmental stages. Total RNA and first-strand cDNA synthesis of the samples were carried out according to Lv *et al.* (2016).

**Table 1:** Primer pairs for qRT-PCR.

	Unigene	TAIR annotation	Abbreviations	Fwd 5’—3’	Rev 5’—3’
sugar biosynthesis	Unigene14855	galactinol—sucrose galactosyltransferase 5	*CaRFS5*	GCCCTACATCTTGCTCCTACC	AACTTTACTGGACCCGCTTTC
	CL2479.Contig12	Sucrose-phosphatase 2	*CaSPP2*	AAACAATATCGTATTTGGGTCG	TATGCAACATCGCCTGTCTTC
	CL444.Contig2	alkaline alpha-galactosidase seed imbibition protein	*CaSIP*	TCCTCTACGAAGCCTAAAC	CGATAATGTCCGTCGAATT
	CL2479.Contig7	sucrose-phosphatase 1	*CaSPP1*	GGGGACTGAAATAACGTATGG	TCTTGAGCCTTGTCTTTGTGA
	CL1440.Contig1	sucrose synthase 7	*CaSUS7*	CTTGACCAACTTCCGCCCTAT	TTCTGAGCCTCTTGTTCTTGC
nectary-enriched genes	Unigene16506	enzyme of the cupin superfamily	*CaCUPIN*	TAACAACCCTCCCGACTCTAA	GGTAAACCCTCACCTTTCCCT
	CL4573.Contig1	acid beta-fructosidase	*CaβFRA*	AAAGGCAGTGAATGGAGCAGC	GATTTCTTGATGGGACGGTGG
	CL2191.Contig2	beta-fructofuranosidase	*CaβFFA*	GGGCCGAGATGGTCATTGGAG	CCGAGTGAAGCGGGTGTTTGG
	CL7013.Contig2	protein CRABS CLAW	*CaCRC*	CCCACTCTTCAGGGTTATTGT	GTGTTTCTTCTCAGGAGGTTT
	CL4219.Contig2	TDR8 protein	*CaTDR8*	CTGAAGTTGCCCTTCTTCTTT	TTAGTCTTCCAAACCTCCACA
	Unigene21647	L-ascorbate oxidase	*CaAAO1*	CCATAGCCGTAACCTTATCCC	CTTATCGCCAACCTTGAGTGA
	CL3588.Contig1	L-ascorbate oxidase	*CaAAO2*	GCGCAGCTAGACCTAACCCTC	TGAACACCTTGTCCGAAACAC
	CL2403.Contig2	L-ascorbate oxidase	*CaAAO3*	ACGGGATACAGAACAGGAGAA	CCACCAGCAGCTTTATGAAAT
	DQ252512		*ACTIN*	TGCAGGAATCCACGAGACTAC	TACCACCACTGAGCACAATGTT

### Phylogenetic analysis

Alignment of the nucleotide sequences was computed using ClusterX, and the phylogenetic trees were created using the ClustalX and Mega 4.0 software packages with standard parameters.

### Putative molecular markers

 Potential simple sequence repeat (SSR) markers were detected using the MISA Perl script (http://pgrc.ipkgatersleben.de/misa/). Mono-, di-, tri-, tetra-, penta- and hexa-nucleotide sequences with minimum repeat numbers of 10, 6, 5, 5, 5, and 5, respectively, were used as the search criteria. SOAPaligner software (Release 2.21.08-13-2009) was used to mine for the single nucleotide polymorphism (SNP) markers. The thresholds for SNP identification were carried out according to Liu *et al.* (2013).

### Statistical analysis

Three biological replicates were generated for each developmental stage (each sample containing flowers from 10 individual plants), with technical triplicates for each sample. An actin gene was chosen as internal control for normalization, and relative gene expression was calculated using the 2^-ΔΔCt^ method.

## Results

### Flower transcriptome sequencing output and *de novo* assembly

By Illumina sequencing 57,826,112, 59,057,626, and 59,459,964 raw reads were generated for the B1, B2, and B3 development stages, respectively. After filtering of low-complexity reads, low-quality reads, and repetitive reads, more than 53 million 49nt clean reads were obtained for the B1, B2, and B3 cDNA libraries, respectively (Table 2).

**Table 2:** Output statistics for pepper flower cDNA libraries.

Sample	Total raw reads	Total clean reads	Total clean nucleotides (nt)	Q20 percentage	N percentage	GC percentage
B1	57826112	53911440	4852029600	97.13%	0.00%	43.11%
B2	59057626	55408880	4986799200	97.37%	0.00%	42.84%
B3	59459964	55459964	4971742380	97.09%	0.00%	42.77%

*Total reads and Total nucleotides are clean reads and clean nucleotides; Total nucleotides should be more than contract provision; Q20 percentage is the proportion of nucleotides with quality value larger than 20; N percentage is the proportion of unknown nucleotides in clean reads; GC percentage is the proportion of guanidine and cytosine nucleotides among total nucleotides. Total Clean Nucleotides = Total Clean Reads1 x Read1 size + Total Clean Reads2 x Read2 size

The Trinity program was used for *de novo* assembly of the high-quality reads. The number of contigs and information about the pepper flower contigs is shown in Table 3. A total of 82,718, 77,061, and 91,491 unigenes, respectively, were assembled, with an average unigene length of 704, 657, and 716 nt. Details about the pepper unigenes are shown in Table 3.

**Table 3:** Statistics of assembly quality for pepper flower.

	Sample	Total number	Total length (nt)	Mean length (nt)	N50	Total consensus sequences	Distinct clusters	Distinct singletons
contigs	B1	147640	50014106	339	633			
	B2	127553	45583704	357	673			
	B3	165374	54415175	329	599			
unigenes	B1	82718	58197759	704	1262	82718	26428	56290
	B2	77061	50601412	657	1137	77061	22788	54273
	B3	91491	65522811	716	1339	91491	29588	61903
	All	97475	93202979	956	1555	97475	39092	58383

The BLASTX program (E-value < 10^-5^) was used to obtain 59,285 significant BLAST hits. The size distribution for the CDSs is shown in Figure S1. When the CDSs of the unigenes did not result in any BLAST hits, they were predicted using ESTScan (Iseli *et al.*, 1999). A total of 2303 unigenes were analyzed using this method (the size distribution of the ESTs is shown in Figure S2).

### Functional annotation

BLASTX was used to search for distinct gene sequences against the Nr database. the hit rate of 61,365 unigenes exceeded the E-value cutoff. Similarly, 37,039 unigenes were identified in the SwissProt database. A total of 76,833 unigenes were annotated using one or more of the databases, suggesting they had relatively well conserved functions.

The unigenes were searched against the COG database in order to predict and classify possible functions. Out of 61,365 Nr hits, 41,683 sequences had COG classifications that were distributed across 25 COG categories (Figure S3). Among the 25 COG categories, "General function prediction only" represented the largest group (7196, 17.3%), followed by "Replication, recombination and repair" (4008, 9.6%), "Transcription" (3806, 9.1%), and "Signal transduction mechanisms" (3132, 7.5%). The smallest groups were "Nuclear structure" (4 unigenes) and "Extracellular structures" (15 unigenes).

BLAST2GO was used to assign 33,8092 unigenes and summarized the terms into three main GO categories and 55 sub-categories (functional groups) (Figure S4). In each of the three main categories of the GO classification system (Biological process, Cellular component, and Molecular function), the dominant terms were "Cellular process", "Metabolic process", "Cell", "Cell part", "Organelle", "Binding", and "Catalytic activity". About half of the genes were in the Biological process category.

The annotated sequences were mapped to the reference canonical pathways contained in the KEGG database. In total, 34,445 unigenes were assigned to 128 KEGG pathways. The most highly represented category was "Metabolic pathways" with 7658 (22.23%) members. The "Biosynthesis of secondary metabolites" and "Plant-pathogen interaction" pathways were also well represented, with 4110 (11.93%) members and 1969 (5.72%) members, respectively.

### Statistical analysis of the differential expression genes (DEGs) during flower development

**Figure 1:**
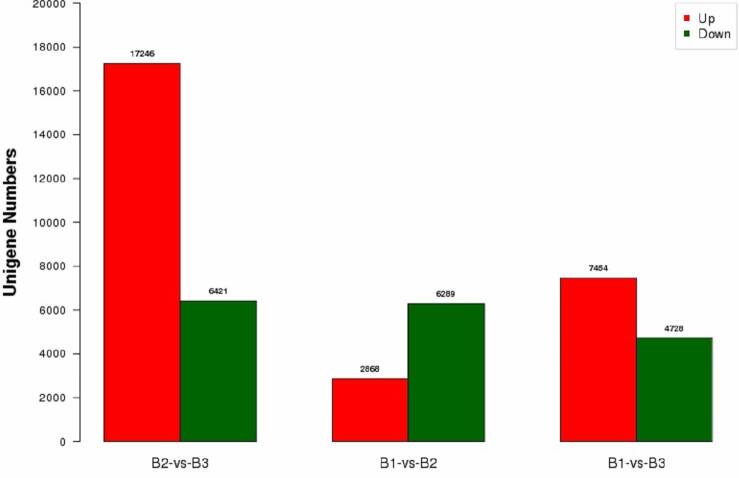
Differentially expressed genes (DGEs) between different developmental stages of flowers.

RNA samples from the B1, B2, and B3 stages were used as the libraries for nectar production and nectary development. Pairs of the three libraries (B2 *vs.* B1, B3 *vs.* B1, and B3 *vs.* B2) were compared. For each different stage, we identified thousands of DEGs, indicating that there had been substantial changes. The B2 *vs.* B1 comparison showed 8,955 DEGs, with 2666 up-regulated and 6289 down-regulated (Figure 1). In the B3 *vs.* B1 comparison, 12,182 DEGs were found, of which 7454 were up-regulated and 4728 down-regulated (Figure 1). A total of 17,246 DEGs were up-regulated and 6421 were down-regulated in the B3 *vs.* B2 comparison (Figure 1). Hence, the number of DEGs in the B2 *vs.* B3 comparison was the largest, and the number of B2 *vs.* B1 differences were the lowest in the three pair comparisons.

Next, we mapped the DEGs to the KEGG databases and compared them to our transcriptome data. A number of genes were significantly enriched at the B3 stage. The B2 *vs.* B1 comparison showed that the most highly represented category was "Metabolic pathways" with 795 (21.75%) members. The "Biosynthesis of secondary metabolites", "RNA transport", and the "Plant-pathogen interaction" pathways were also well represented, with 495 (12.54%) members, 255 (6.97%) members, and 239 (6.53%) members, respectively (Figure S5). In the B3 *vs.* B1 comparison, the most highly represented category was "Metabolic pathways", with 1180 (25.11%) members. The "Biosynthesis of secondary metabolites" and "Plant-pathogen interaction" pathways were also well represented, with 693 (14.75%) members and 370 (7.87%) members, respectively (Figure S6). In the B3 *vs.* B2 comparison, the most highly represented category was "Metabolic pathways", with 1872 (23.45%) members. The "Biosynthesis of secondary metabolites" and "Plant-pathogen interaction" pathways were also well represented, with 1069 (13.39%) members and 583 (7.3%) members, respectively (Figure S7).

### Analysis of nectar biosynthesis associated genes and qPCR validation

Sugars are the principal solutes in most nectars. Genes involved in sugar metabolism were expected to be well represented within the flower transcriptome, which was the case in this study. A total of 18 sugar metabolizing and modifying genes were identified from the flower transcriptome (Table S1), and 17 of those unigenes belonged to four genes families, which were *sucrose-phosphatase 2* (*CaSPP2*) (four unigenes), *alkaline alpha-galactosidase* (*CaAGA*) (four unigenes), *sucrose-phosphatase 1* (*CaSPP1*) (three unigenes), and *sucrose synthase 7-like* (*CaSUS7*) (six unigenes). The last unigene was *galactinol-sucrose galactosyltransferase 5-like* (*CaGSG5*). The B2 *vs.* B1 libraries comparison showed that seven unigenes were up-regulated and eight were down-regulated (Table S2). In the B3 *vs.* B1 comparison, 15 unigenes were up-regulated and one was down-regulated (Table S3), whereas 13 unigenes were up-regulated and no unigenes were down-regulated in the B3 vs B2 comparison (Table S4).

**Figure 2:**
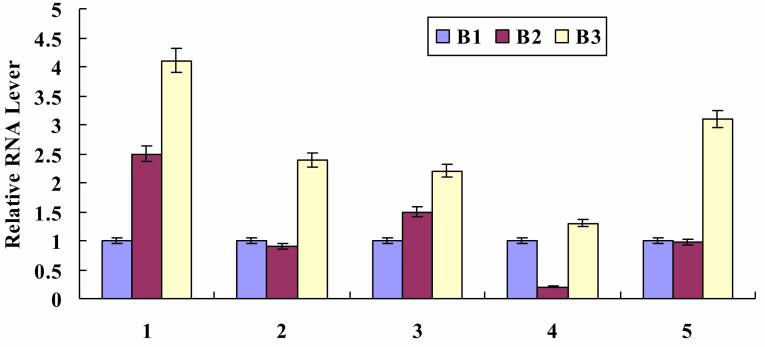
Real time PCR analysis of selected genes involved in sucrose biosysnthesis. 1: Unigene14855_All; 2: CL2479.Contig12_All; 3: CL444.Contig2_All; 4: CL2479.Contig7_All; 5: CL1440.Contig1_All.

In the sugar metabolism category, five unigenes were investigated further. In general, the identification of the genes with different expressions that are putatively associated with sucrose biosynthesis are summarized in Figure 2. For all the selected unigenes, most of their expressions continuously increased and peaked at the nectar production stage (B3).

### Analysis of nectary development association genes and qPCR validation

Forty seven known *Arabidopsis* nectary-enriched unigenes were identified from the pepper flower transcriptomes (Table S5). Two belonged to the transcription factor *CRC*, two were cupin family proteins (*CUPIN*), eight unigenes were part of putative beta-fructosidase (βFRA), one unigene belonged to agamous-like MADS box protein *AGL5*, and the other unigenes belonged to the L-ascorbate oxidase (*AO*). The B2 *vs.* B1 library comparison showed that 20 unigenes were up-regulated and eight were down-regulated (Table S6). In the B3 *vs.* B1 comparison, 26 unigenes were up-regulated and four were down-regulated (Table S7); and in the B3 *vs.* B2 comparison, 35 unigenes were up-regulated and three were down-regulated (Table S8).

**Figure 3:**
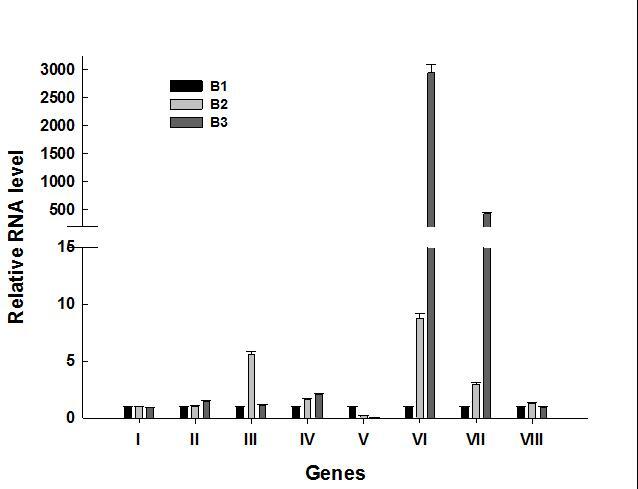
Real time PCR analysis of selected genes involved in nectary development. I: Unigene16506_All; II: CL4573.Contig1_All; III: CL2191. Contig2_All; IV: CL7013.Contig2_All; V: CL4219.Contig2_All; VI: Unigene21647_All; VII: CL3588.Contig1_All; VIII: CL4586.Contig1_All.

Some known *Arabidopsis* nectary-enriched genes were used to select eight unigenes for qPCR analysis. Unigene16506 belongs to the cupin family protein group, and was named *CaCUPIN*. It had a lower expression in B1, was up-regulated in B2, and then down-regulated in B3 samples. CL4573.Contig1 (beta-fructosidase, named *CaβFRA*), CL7013.Contig2 (transcription factor *CRC*, named *CaCRC*), Unigene21647, and CL3588.Contig1 and CL2403.Contig1 (L-ascorbate oxidase, named *CaAO1*, *CaAO2*, and *CaAO3*) were up-regulated in their expression at the flower development stage (B3). The CL2191.Contig2 (beta-fructofuranosidase, named *CaβFFA*) and CL4219. Contig2 (agamous-like MADS box protein *AGL5*, named *CaAGL5*) displayed similar expression patterns, and were up-regulated at the nectary forming stage (B2) (Figure 3).

### Putative molecular markers

A total of 11,654 SSRs were identified in 97,475 unigenes. Mono-nucleotide SSRs represented the largest fraction (35.9%; 4180), followed by tri-nucleotides (34.7%; 4044), di-nucleotides (24.0%; 2802), hexa-nucleotide tandem repeats (2.3%; 271), penta-nucleotides (0.18%; 207), and tetra-nucleotides (0.13%; 150) (Table 4). Additionally, a total of 17,068, 14,407, and 20,350 putative SNPs were detected in the B1, B2, and B3 libraries, respectively (Table 5).

**Table 4:** Distribution of SSRs identified using MISA software.

Number of repeats	Mono-nucleotide repeats	Di-nucleotide repeats	Tri-nucleotide repeats	Quad-nucleotide repeat	Penta-nucleotide repeats	Hexa-nucleotide repeats
4	0	0	0	0	162	269
5	0	0	2,299	116	45	0
6	0	1,215	1,049	34	0	0
7	0	651	620	0	0	2
8	0	353	76	0	0	0
9	0	217	0	0	0	0
10	0	157	0	0	0	0
11	0	194	0	0	0	0
12	1,129	15	0	0	0	0
13	700	0	0	0	0	0
14	532	0	0	0	0	0
15	315	0	0	0	0	0
16	220	0	0	0	0	0
17	157	0	0	0	0	0
18	162	0	0	0	0	0
19	209	0	0	0	0	0
20	275	0	0	0	0	0
21	238	0	0	0	0	0
22	161	0	0	0	0	0
23	78	0	0	0	0	0
24	4	0	0	0	0	0
Subbotal	4,180	2,802	4,044	150	207	271

**Table 5:** Distribution of SNPs identified using SOAPaligner software.

SNP Type	B1	B2	B3
Transition	11,219	9,422	13,306
A-G	5,826	4,891	6,889
C-T	5,393	4,531	6,417
Transversion	5,849	4,985	7,044
A-C	1,566	1,296	1,845
A-T	1,622	1,463	2,047
C-G	1,029	879	1,211
G-T	1,632	1,347	1,941
Total	17,068	14,407	20,350

## Discussion

### Global gene expression patterns in pepper flowers

High-throughput mRNA sequencing technology is highly suitable for gene expression profiling in non-model organisms. Before this study, most pepper sequence studies were based on EST sequencing, very few tags had been reported in public databases, and there was little available genetic or genomic information. This study used RNA-Seq technology on the Illumina HiSeqTM 2000 platform to profile the pepper flower transcriptomes. We obtained 53,911,440, 55,408,880, and 55,241,582 clean reads, and identified 82,718, 77,061 and 91,491 unigenes from *de novo* assembly in the B1, B2, and B3 libraries, respectively. The gene or protein names and descriptions were assessed, and their putative conserved domains, gene ontology terms, and potential metabolic pathways were annotated. This study will help to improve our understanding of the processes involved in regulating flower development, nectar biosynthesis, and nectary development in pepper flowers. More contigs and unigenes were reported compared to previous transcriptomic studies in other plants, such as tomato (Pandurangaiah *et al.*, 2016), potato (Cao *et al.*, 2016), and eggplant (Ramesh *et al.*, 2016), which indicated that pepper contains abundant gene resources. We believe that our data will help to provide important new insights and facilitate further studies on pepper genes and their functions.

### Nectar biosynthesis association genes

**Figure 4:**
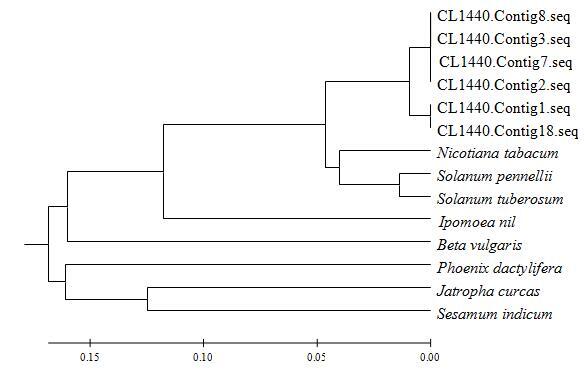
Phylogenetic tree of sucrose synthase 7. *Nicotiana tabacum*: XM_016585183; *Solanum pennellii*: XM_015210288; *Solanum tuberosum*: XM_006348118; *Ipomoea nil*: XM_019321835; *Beta vulgaris*: XM_010676936; *Phoenix dactylifera*: XM_008806164; *Jatropha curcas*: XM_012220325; *Sesamum indicum*: XM_011088670

As we analyzed progressive flowering stages, not surprisingly a large number of genes associated with sugar metabolism and processing were found differentially expressed. This is in agreement with the hypothesis that simple sugars are the principal solutes in most nectars (Davis *et al.*, 1998; Baum *et al.*, 2001; Pacini *et al.*, 2007; Ren *et al.*, 2007). Sugar modifying enzymes and sugar transporters control sugar transport and metabolism in plant cells and tissues. They also play key roles in establishing and maintaining sugar concentrations across membranes (Roitsch, 1999). For example, sucrose synthase (SUS, EC 2.4.1.13) is a known glycosyltransferase in plants. The enzyme catalyzes the reversible transfer of a glucosyl moiety between fructose and a nucleoside diphosphate (NDP) (NDP-glucose + fructose ↔ sucrose + NDP) (Diricks *et al.*, 2015). In this study, *CaSUS7* (sucrose synthase 7-like family proteins: CL1440.Contig1, CL1440.Contig2, CL1440.Contig3, CL1440.Contig7, CL1440.Contig8, and CL1440.Contig18) were highly homologous to *Nicotiana tabacum* (XM_016585183), *Solanum pennellii* (XM_015210288), *S. tuberosum* (XM_006348118), *Ipomoea nil* (XM_019321835), *Beta vulgaris* (XM_010676936), *Phoenix dactylifera* (XM_008806164), *Jatropha curcas* (XM_012220325), and *Sesamum indicum* (XM_011088670) (Figure 4) which strongly suggests that they all might encode *SUS7* proteins that catalyze the reversible reaction of fructose to a nucleoside diphosphate. *SUS* has been shown to play a crucial role in nectar production (Kram *et al*., 2009). All of the genes associated with *CaSUS* were strongly up-regulated, which indicates that *CaSUS* may play a crucial role in pepper nectar production.

In addition to *CaSUS*, we identified a number of other genes that were up-regulated at the flower development and were involved in simple sugar metabolism. These were *CaGSG5* (Unigene14855), 4 *CaS6PP* (CL2479.Contig12, CL2479.Contig11, CL2479.Contig5, and CL2479.Contig2), 3 *CaSPP1* (CL2479.Contig1, CL2479.Contig6, and CL2479.Contig7), 4 *CaGSG1* (CL444.Contig4, CL444.Contig1, CL444.Contig2, and CL444.Contig5). Significantly, the TAIR AraCyc database (http://www.arabidopsis.org/biocyc/index.jsp) suggested that these genes can be tentatively assigned functions in sucrose metabolism (synthesis/degradation). These results showed that the canonical sucrose biosynthesis pathway was represented by genes that were differentially expressed within the flower at the nectar producing stage (Figure S5).

### Nectary development association genes

**Figure 5:**
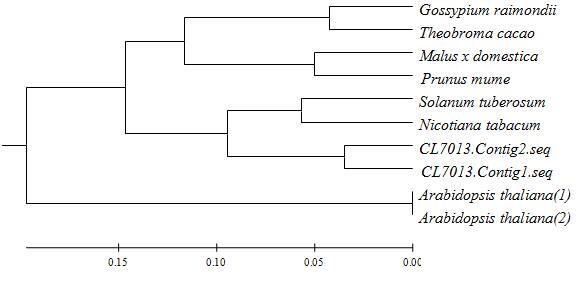
Phylogenetic tree of *CRC. Arabidopsis thaliana*(1): BT008618; *Arabidopsis thaliana*(2): DQ446412; *Gossypium raimondii*: XM_012625767; *Malus x domestica*: XM_008391173; *Nicotiana tabacum*: AY854799; *Prunus mume*: XM_008245598; *Solanum tuberosum*: XM_006348604; *Theobroma cacao*: XM_018114782

Transcription process genes were also highly represented within nectary expressed genes. Forty-seven unigenes showed open flower (nectary-enriched) expression profiles (Table-S5). There were six members of the *YABBY* transcription factor gene families in *Arabidopsis* (*CRC*, *FILAMENTOUS FLOWER*, *YABBY3*, *INNER NO OUTER*, *YABBY*2, and *YABBY5*) and they are determinants of abaxial cell fate in the lateral floral organs (Siegfried *et al*., 1999). *CRABS CLAW* (At1g69180, *CRC*) encodes a transcription factor associated with the regulation of carpel and nectary development (Alvarez and Smyth, 1999). *CRC* is currently the only known gene to be absolutely required for nectary development (Lee *et al.*, 2005b; Alvarez and Smyth, 1999). This study identified two *CaCRC*s that were highly homologous to *N. tabacum* (AY854799), *S. tuberosum* (XM_006348604), Malus x *domestica* (XM_008391173), *Prunus mume* (XM_008245598), *Gossypium raimondii* (XM_012625767), *Theobroma cacao* (XM_018114782), and *A. thaliana* At1g69180 (BT008618, DQ446412) (Figure 5). Both were differentially expressed at the pepper nectary forming stage, which suggests that they may be involved in nectary development or function.

## Supplementary material

The following online material is available for this article:

Figure S1 - Size distribution of CDSs produced by searching unigene sequences against various protein databases (Nr, SwissProt, KEGG, and COG, in order) using BLASTX (E-value 10^-5^).

Figure S2 - Size distribution of ESTs obtained from the ESTScan results.

Figure S3 - COG function classification of the unigenes.

Figure S4 - GO function classification of the unigenes.

Figure S5 - Histogram of the GO classifications for genes that were differentially expressed between B2 and B1.

Figure S6 - Histogram of the GO classifications for genes that were differentially expressed between B3 and B1.

Figure S7 - Histogram of the G O classifications for genes that were differentially expressed between B3 and B2

Table S1 - Sugar metabolism unigenes derived from the flower transcriptomes

Table S2 - Sugar metabolism unigenes expression comparison between stages B2 and B1.

Table S3 - Sugar metabolism unigenes expression comparison between stages B3 and B1.

Table S4 - Sugar metabolism unigenes expression comparison between stages B3 and B2.

Table S5 - Nectary enriched unigenes derived from the flower transcriptomes.

Table S6 - Nectary-enriched unigenes expression comparison between stages B2 and B1.

Table S7 - Nectary-enriched unigenes expression comparison between stages B3 and B1.

Table S8 - Nectary-enriched unigenes expression comparison between stages B3 and B2.
