# Supplementary material for: Flower transcriptome dynamics during nectary development in pepper (Capsicum annuum L.)
Source: Genet Mol Biol. 2020 May 29;43(2):e20180267. doi: 10.1590/1678-4685-GMB-2018-0267 (PMC7263202; doi:10.1590/1678-4685-GMB-2018-0267)
Supplement: Figure S1 - [file 1415-4757-GMB-43-2-e20180267-s1.pdf]

Supplementary Material to "Flower transcriptome dynamics during nectary development in pepper (*Capsicum annuum L.*)"

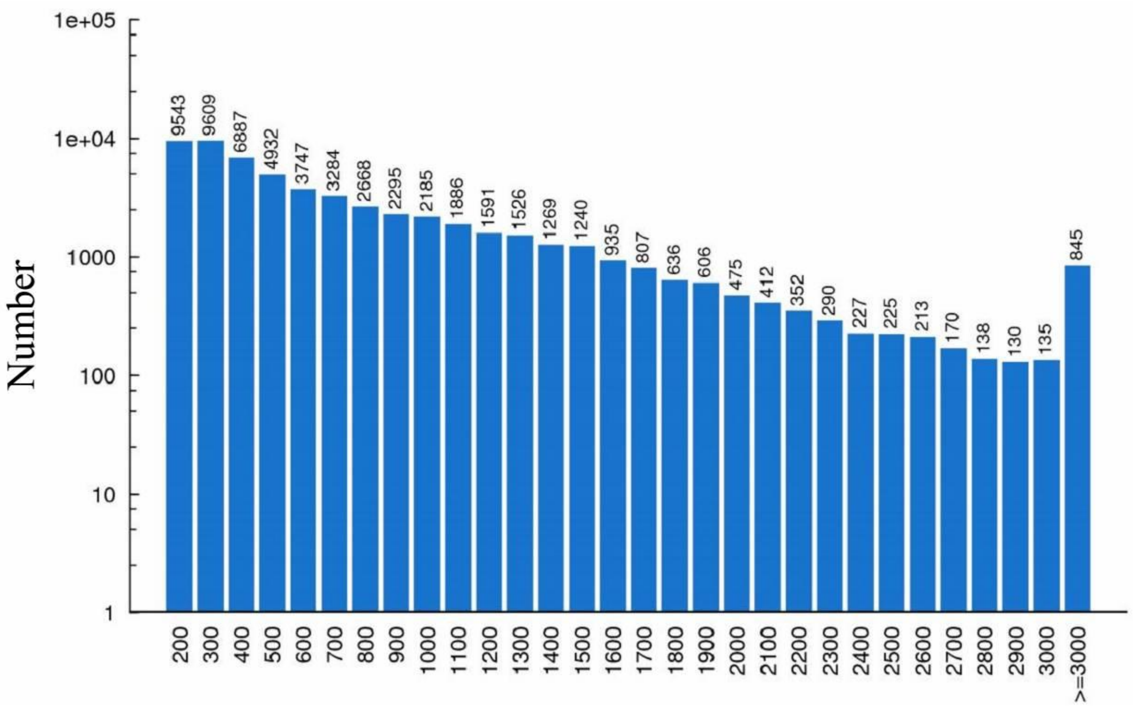

Figure S1 - Size distribution of CDSs produced by searching unigene sequences against various protein databases (Nr, SwissProt, KEGG, and COG, in order) using BLASTX (E-value 10^{-5}).
