# Supplementary material for: Flower transcriptome dynamics during nectary development in pepper (Capsicum annuum L.)
Source: Genet Mol Biol. 2020 May 29;43(2):e20180267. doi: 10.1590/1678-4685-GMB-2018-0267 (PMC7263202; doi:10.1590/1678-4685-GMB-2018-0267)
Supplement: Figure S3 - [file 1415-4757-GMB-43-2-e20180267-s3.pdf]

Supplementary Material to "Flower transcriptome dynamics during nectary development in pepper (*Capsicum annuum L.*)"

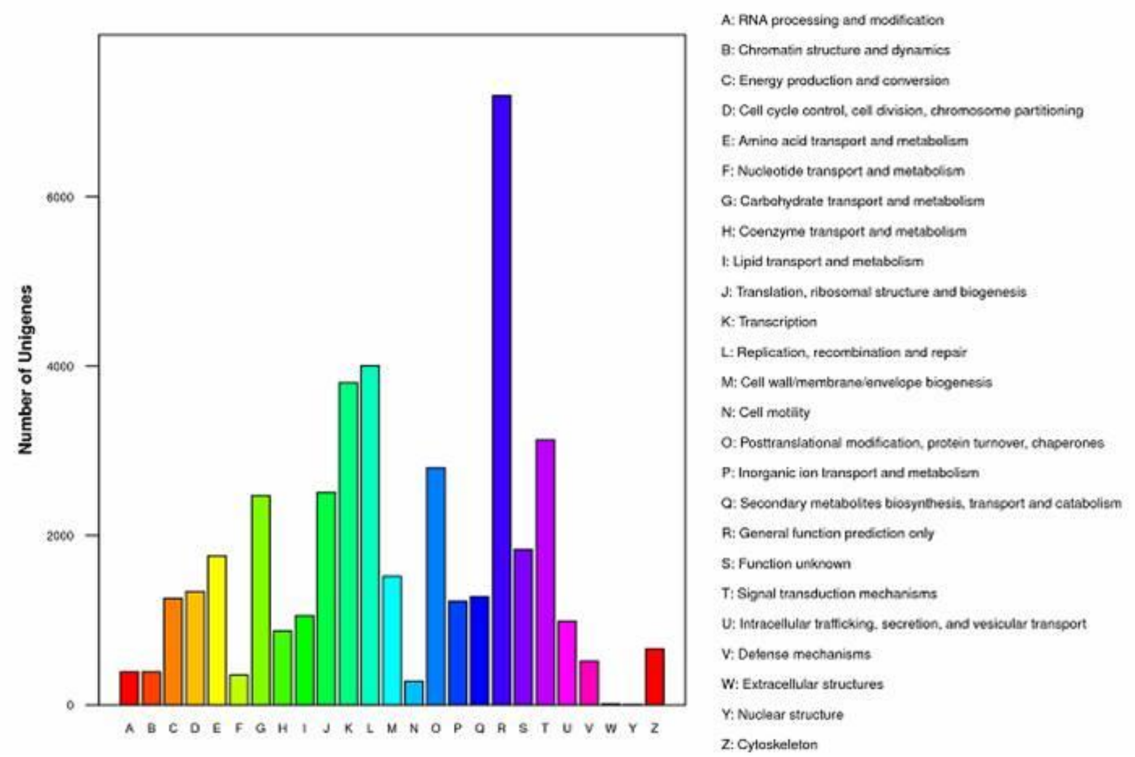

Figure S3 - COG function classification of the unigenes.
