# Supplementary material for: Flower transcriptome dynamics during nectary development in pepper (Capsicum annuum L.)
Source: Genet Mol Biol. 2020 May 29;43(2):e20180267. doi: 10.1590/1678-4685-GMB-2018-0267 (PMC7263202; doi:10.1590/1678-4685-GMB-2018-0267)
Supplement: Table S1 - [file 1415-4757-GMB-43-2-e20180267-s8.pdf]

Genetics and Molecular Biology 43, 2, (2020)

Supplementary Material to "Flower transcriptome dynamics during nectary development in pepper (Capsicum annuum L.)"

Table S1 - Sugar metabolism unigenes from flower transcriptomes.

geneID	Gene Length	B1_raw fragments	B1_FPKM	B2_raw fragments	B2_FPKM	B3_raw fragments	B3_FPKM
Unigene14855	2743	157	3.1694	358	6.9123	632	12.2723
CL2479.Contig12	1964	32	0.9022	28	0.7551	76	2.0611
CL444.Contig4	899	19	1.1703	35	2.0619	27	1.5997
CL444.Contig5	1002	28	1.5474	41	2.1671	22	1.1695
CL2479.Contig5	2262	26	0.6365	23	0.5385	35	0.8242
CL444.Contig2	2569	18	0.388	25	0.5154	39	0.8086
CL2479.Contig7	1457	15	0.5701	3	0.1091	19	0.6946
CL1440.Contig2	1994	9	0.2499	12	0.3187	11	0.2938
CL2479.Contig2	2144	9	0.2324	5	0.1235	10	0.2484
CL1440.Contig8	1968	3	0.0844	1	0.0269	9	0.2436
CL2479.Contig6	1827	8	0.2425	4	0.116	7	0.2041
CL2479.Contig1	1602	16	0.553	8	0.2645	5	0.1662
CL1440.Contig18	1976	5	0.1401	1	0.0268	6	0.1617
CL1440.Contig7	1890	3	0.0879	3	0.0841	4	0.1127
CL1440.Contig3	1981	2	0.0559	3	0.0802	4	0.1076
CL444.Contig1	2477	1	0.0224	5	0.1069	4	0.086
CL1440.Contig1	1901	1	0.0291	1	0.0279	3	0.0841
CL2479.Contig11	2010	1	0.0275	0	0	2	0.053
