# Supplementary material for: Flower transcriptome dynamics during nectary development in pepper (Capsicum annuum L.)
Source: Genet Mol Biol. 2020 May 29;43(2):e20180267. doi: 10.1590/1678-4685-GMB-2018-0267 (PMC7263202; doi:10.1590/1678-4685-GMB-2018-0267)
Supplement: Table S2 - [file 1415-4757-GMB-43-2-e20180267-s9.pdf]

Genetics and Molecular Biology 43, 2, (2020)

Supplementary Material to "Flower transcriptome dynamics during nectary development in pepper (Capsicum annuum L.)"

Table S2 - Sugar metabolism unigenes expression in B2-vs-B1.

geneID	Gene Length	B1_raw fragments	B2_raw fragments	B1_FPKM	B2_FPKM	log2 Ratio (B2/B1)	Up-Down-Regulation(B2/B1)	P-value	FDR
CL4827.Contig1	7295	6091	3254	46.2347	23.6244	-0.9687	Down	9.61E-221	4.19E-218
Unigene14855	2743	157	358	3.1694	6.9123	1.124956	Up	2.53E-17	6.84E-16
CL7371.Contig2	1114	115	35	5.7163	1.664	-1.78043	Down	3.81E-12	7.08E-11
CL4827.Contig2	210	9	2	2.3732	0.5044	-2.23419	Down	0.031938	0.086719
CL444.Contig4	899	19	35	1.1703	2.0619	0.817096	Up	0.044949	0.113
CL444.Contig5	1002	28	41	1.5474	2.1671	0.48592	Up	0.170656	0.305949
CL444.Contig2	2569	18	25	0.388	0.5154	0.409636	Up	0.364244	0.523549
CL2479.Contig7	1457	15	3	0.5701	0.1091	-2.38556	Down	0.003271	0.01353
CL1440.Contig2	1994	9	12	0.2499	0.3187	0.350848	Up	0.594318	0.718982
CL2479.Contig2	2144	9	5	0.2324	0.1235	-0.9121	Down	0.262768	0.413926
CL1440.Contig8	1968	3	1	0.0844	0.0269	-1.64964	Down	0.347796	0.505958
CL2479.Contig6	1827	8	4	0.2425	0.116	-1.06386	Down	0.23342	0.385351
CL2479.Contig1	1602	16	8	0.553	0.2645	-1.06401	Down	0.085449	0.183695
CL1440.Contig18	1976	5	1	0.1401	0.0268	-2.38615	Down	0.111031	0.223868
CL1440.Contig3	1981	2	3	0.0559	0.0802	0.520754	Up	0.729686	0.823036
CL444.Contig1	2477	1	5	0.0224	0.1069	2.254691	Up	0.14027	0.26373
CL2479.Contig11	2010	1	0	0.0275	0	-4.78136	Down	0.47799	0.633474
