# Supplementary material for: Flower transcriptome dynamics during nectary development in pepper (Capsicum annuum L.)
Source: Genet Mol Biol. 2020 May 29;43(2):e20180267. doi: 10.1590/1678-4685-GMB-2018-0267 (PMC7263202; doi:10.1590/1678-4685-GMB-2018-0267)
Supplement: Table S3 - [file 1415-4757-GMB-43-2-e20180267-s10.pdf]

Genetics and Molecular Biology 43, 2, (2020)

Supplementary Material to “Flower transcriptome dynamics during nectary development in pepper (Capsicum annuum L.)”

Table S3 - Sugar metabolism unigenes expression in B3-vs-B1.

geneID	Gene Length	B1_raw fragments	B3_raw fragments	B1_FPKM	B3_FPKM	log2 Ratio (B3/B1)	Up-Down-Regulation (B3/B1)	P-value	FDR
CL4827.Contig1	7295	6091	9260	46.2347	67.6116	0.548295	Up	8.59E-120	8.16E-118
Unigene14855	2743	157	632	3.1694	12.2723	1.953124	Up	1.58E-64	8.42E-63
CL7371.Contig2	1114	115	127	5.7163	6.0723	0.087161	Up	0.640696	0.763096
CL2479.Contig12	1964	32	76	0.9022	2.0611	1.191895	Up	4.67E-05	0.000268
CL444.Contig4	899	19	27	1.1703	1.5997	0.450923	Up	0.301078	0.459265
CL2479.Contig5	2262	26	35	0.6365	0.8242	0.372834	Up	0.322072	0.481476
CL444.Contig2	2569	18	39	0.388	0.8086	1.05937	Up	0.008338	0.026958
CL2479.Contig7	1457	15	19	0.5701	0.6946	0.284967	Up	0.575746	0.710507
CL1440.Contig2	1994	9	11	0.2499	0.2938	0.233483	Up	0.730146	0.827093
CL2479.Contig2	2144	9	10	0.2324	0.2484	0.096055	Up	0.89282	0.945653
CL1440.Contig8	1968	3	9	0.0844	0.2436	1.529199	Up	0.106655	0.214754
CL2479.Contig1	1602	16	5	0.553	0.1662	-1.73436	Down	0.013173	0.03976
CL1440.Contig18	1976	5	6	0.1401	0.1617	0.206863	Up	0.827458	0.894025
CL1440.Contig7	1890	3	4	0.0879	0.1127	0.358552	Up	0.769438	0.851765
CL1440.Contig3	1981	2	4	0.0559	0.1076	0.944758	Up	0.485598	0.637465
CL444.Contig1	2477	1	4	0.0224	0.086	1.940838	Up	0.23749	0.389114
CL1440.Contig1	1901	1	3	0.0291	0.0841	1.531087	Up	0.399746	0.563723
CL2479.Contig11	2010	1	2	0.0275	0.053	0.946561	Up	0.654408	0.772727
