# Supplementary material for: Flower transcriptome dynamics during nectary development in pepper (Capsicum annuum L.)
Source: Genet Mol Biol. 2020 May 29;43(2):e20180267. doi: 10.1590/1678-4685-GMB-2018-0267 (PMC7263202; doi:10.1590/1678-4685-GMB-2018-0267)
Supplement: Table S4 - [file 1415-4757-GMB-43-2-e20180267-s11.pdf]

Genetics and Molecular Biology 43, 2, (2020)

Supplementary Material to "Flower transcriptome dynamics during nectary development in pepper (Capsicum annuum L.)"

Table S4 - Sugar metabolism unigenes expression in B3-vs-B2.

geneID	Gene Length	B2_raw fragments	B3_raw fragments	B2_FPKM	B3_FPKM	log2 Ratio (B3/B2)	Up-Down-Regulation(B3/B2)	P-value	FDR
CL4827.Contig1	7295	3254	9260	23.6244	67.6116	1.516993099	Up	0	0
Unigene14855	2743	358	632	6.9123	12.2723	0.828167917	Up	8.83E-19	1.18E-17
CL7371.Contig2	1114	35	127	1.664	6.0723	1.867587635	Up	9.00E-14	8.85E-13
CL2479.Contig12	1964	28	76	0.7551	2.0611	1.448674881	Up	1.61E-06	7.65E-06
CL4827.Contig2	210	2	8	0.5044	2.0291	2.008199786	Up	0.064068	0.110889
CL2479.Contig5	2262	23	35	0.5385	0.8242	0.614048119	Up	0.112447	0.179535
CL444.Contig2	2569	25	39	0.5154	0.8086	0.649733666	Up	0.077746	0.130346
CL2479.Contig7	1457	3	19	0.1091	0.6946	2.670531309	Up	0.000465	0.001418
CL7662.Contig2	724	5	7	0.3658	0.515	0.493517357	Up	0.573934	0.662932
CL2479.Contig2	2144	5	10	0.1235	0.2484	1.008154132	Up	0.205978	0.297269
CL1440.Contig8	1968	1	9	0.0269	0.2436	3.178836055	Up	0.011417	0.024821
CL2479.Contig6	1827	4	7	0.116	0.2041	0.815151377	Up	0.382226	0.4858
CL1440.Contig18	1976	1	6	0.0268	0.1617	2.593014773	Up	0.069078	0.117814
CL1440.Contig7	1890	3	4	0.0841	0.1127	0.42230981	Up	0.720358	0.786483
CL1440.Contig3	1981	3	4	0.0802	0.1076	0.424003936	Up	0.720358	0.787748
CL1440.Contig1	1901	1	3	0.0279	0.0841	1.591840678	Up	0.37146	0.477384
CL2479.Contig11	2010	0	2	0	0.053	5.727920455	Up	0.247876	0.345247
