# Supplementary material for: Flower transcriptome dynamics during nectary development in pepper (Capsicum annuum L.)
Source: Genet Mol Biol. 2020 May 29;43(2):e20180267. doi: 10.1590/1678-4685-GMB-2018-0267 (PMC7263202; doi:10.1590/1678-4685-GMB-2018-0267)
Supplement: Table S5 - [file 1415-4757-GMB-43-2-e20180267-s12.pdf]

Genetics and Molecular Biology 43, 2, (2020)

Supplementary Material to “Flower transcriptome dynamics during nectary development in pepper (Capsicum annuum L.)”

Table S5 - Nectary enriched unigenes from flower transcriptomes.

Gene family	geneID	gene length	B1_rawfragments	B1_FPKM	B2_rawfragments	B2_FPKM	B3_rawfragments	B3_FPKM
cupin	Unigene16506	887	1227	76.5995	1299	77.5628	1149	68.9973
	Unigene16525	893	357	22.1372	257	15.2423	198	11.81
beta-fructosidase	CL4573.Contig1	2326	2752	65.5154	2979	67.8311	4238	97.0481
	CL4573.Contig2	2155	42	1.0792	62	1.5237	551	13.6188
	Unigene1441	440	19	2.3911	17	2.0463	14	1.6948
	CL2191.Contig2	1885	48	1.4101	280	7.8671	56	1.5824
	CL2191.Contig6	1953	1	0.0284	1	0.0271	13	0.3545
	CL2191.Contig1	1847	2	0.06	5	0.1434	7	0.2019
	CL2191.Contig5	2039	0	0	3	0.0779	6	0.1567
	CL7021.Contig6	2752	11	0.2213	14	0.2694	8	0.1548
multi - copper oxidase	Unigene21647	1962	8	0.2258	73	1.9706	24483	664.6628
	CL3588.Contig1	2108	37	0.9719	114	2.8642	16445	415.5267
	CL2403.Contig2	960	1118	64.4875	699	38.5633	2283	126.669
	Unigene11619	2211	3928	98.3757	2393	57.3221	2639	63.575
	Unigene11619	2211	3928	98.3757	2393	57.3221	2639	63.575
	CL4586.Contig1	2150	1387	35.7226	1880	46.3114	1381	34.213
	CL2829.Contig1	2012	1415	38.9434	1298	34.1676	1070	28.3264
	CL5139.Contig1	1825	261	7.9192	401	11.6372	854	24.9247
	Unigene11984	1239	858	38.3461	259	11.0712	518	22.2687
	CL1363.Contig2	2093	310	8.2016	358	9.059	764	19.4428

Gene family	geneID	gene length	B1_rawfragments	B1_FPKM	B2_rawfragments	B2_FPKM	B3_rawfragments	B3_FPKM
	Unigene32629	2067	362	9.6978	396	10.1467	599	15.4355
	CL2829.Contig2	1942	520	14.8272	339	9.2452	548	15.0303
	Unigene32314	2553	675	14.6406	614	12.7375	714	14.8965
	CL7632.Contig1	1759	167	5.2572	156	4.6971	429	12.9905
	CL8488.Contig2	1952	206	5.8438	194	5.2637	410	11.1877
	CL2403.Contig1	292	40	7.5855	21	3.8089	46	8.3909
	Unigene32128	2061	337	9.0543	176	4.5228	280	7.2363
	CL2403.Contig4	1872	123	3.6383	116	3.2819	220	6.2597
	Unigene29898	362	14	2.1415	13	1.902	31	4.5613
	Unigene18601	520	12	1.2779	4	0.4074	32	3.2778
	CL1363.Contig1	202	8	2.193	6	1.5731	12	3.1642
	CL1363.Contig3	1828	103	3.1201	137	3.9693	99	2.8847
	CL4586.Contig2	1570	42	1.4813	30	1.012	54	1.832
	Unigene16391	647	25	2.1396	9	0.7367	20	1.6465
	CL5139.Contig2	847	10	0.6538	8	0.5002	21	1.3206
	Unigene23763	985	80	4.4974	52	2.796	22	1.1897
	CL1363.Contig4	824	15	1.008	18	1.1569	12	0.7757
	CL2157.Contig6	2212	20	0.5007	4	0.0958	5	0.1204
	CL2157.Contig2	2048	3	0.0811	6	0.1552	4	0.104
	CL2157.Contig3	2129	3	0.078	1	0.0249	1	0.025
	CL2157.Contig1	1641	0	0	1	0.0323	0	0
	CL2157.Contig4	2030	0	0	2	0.0522	0	0
	CL2157.Contig5	1949	0	0	1	0.0272	0	0
	CL8488.Contig1	397	0	0	0	0	0	0
claw	CL7013.Contig1	808	1541	105.6079	1513	99.1735	1813	119.5149
	CL7013.Contig2	841	801	52.7402	1395	87.8509	1749	110.7719
agl5	CL4219.Contig2	790	16	1.1215	4	0.2682	1	0.0674
