# Supplementary material for: Flower transcriptome dynamics during nectary development in pepper (Capsicum annuum L.)
Source: Genet Mol Biol. 2020 May 29;43(2):e20180267. doi: 10.1590/1678-4685-GMB-2018-0267 (PMC7263202; doi:10.1590/1678-4685-GMB-2018-0267)
Supplement: Table S6 - [file 1415-4757-GMB-43-2-e20180267-s13.pdf]

Genetics and Molecular Biology 43, 0, (2020)

Supplementary Material to "Flower transcriptome dynamics during nectary development in pepper (Capsicum annuum L.)"

Table S6 - Nectary-enriched unigenes expression in B2-vs-B1.

Gene family	geneID	Gene Length	B1_raw fragments	B2_raw fragments	B1_FPKM	B2_FPKM	log2 Ratio(B2/B1)	Up-Down-Regulation(B2/B1)	P-value	FDR
cupin	Unigene16506	887	1227	1299	76.5995	77.5628	0.01803	Up	0.754054	0.837005
beta-fructosidase	CL4573.Contig1	2326	2752	2979	65.5154	67.8311	0.050113	Up	0.189016	0.331333
	CL4573.Contig2	2155	42	62	1.0792	1.5237	0.497617	Up	0.083932	0.181108
	CL2191.Contig2	1885	48	280	1.4101	7.8671	2.480034	Up	5.36E-39	3.40E-37
	CL2191.Contig1	1847	2	5	0.06	0.1434	1.257011	Up	0.319256	0.477612
	CL2191.Contig5	2039	0	3	0	0.0779	6.283551	Up	0.136506	0.260085
	CL7021.Contig6	2752	11	14	0.2213	0.2694	0.283746	Up	0.636534	0.756536
multi - copper oxidase	Unigene21647	1962	8	73	0.2258	1.9706	3.125518	Up	7.05E-14	1.50E-12
	CL3588.Contig1	2108	37	114	0.9719	2.8642	1.559252	Up	9.28E-10	1.37E-08
	CL4586.Contig1	2150	1387	1880	35.7226	46.3114	0.37453	Up	1.70E-13	3.52E-12
	CL5139.Contig1	1825	261	401	7.9192	11.6372	0.555317	Up	1.03E-06	1.01E-05
	Unigene11984	1239	858	259	38.3461	11.0712	-1.79227	Down	1.74E-81	2.57E-79
	CL1363.Contig2	2093	310	358	8.2016	9.059	0.143446	Up	0.20031	0.343571
	Unigene32629	2067	362	396	9.6978	10.1467	0.065281	Up	0.534702	0.670663
	CL2403.Contig1	292	40	21	7.5855	3.8089	-0.99387	Down	0.009218	0.032151
	Unigene32128	2061	337	176	9.0543	4.5228	-1.00139	Down	1.84E-14	4.09E-13
	Unigene18601	520	12	4	1.2779	0.4074	-1.64926	Down	0.039428	0.101705
	CL1363.Contig3	1828	103	137	3.1201	3.9693	0.347292	Up	0.064639	0.147319
	Unigene16391	647	25	9	2.1396	0.7367	-1.53819	Down	0.003992	0.016062
	CL1363.Contig4	824	15	18	1.008	1.1569	0.198769	Up	0.701826	0.8007

Gene family	geneID	Gene Length	B1_raw fragments	B2_raw fragments	B1_FPKM	B2_FPKM	log2 Ratio(B2/B1)	Up-Down-Regulation(B2/B1)	P-value	FDR
	CL2157.Contig6	2212	20	4	0.5007	0.0958	-2.38585	Down	0.000615	0.003241
	CL2157.Contig2	2048	3	6	0.0811	0.1552	0.936355	Up	0.381486	0.54049
	CL2157.Contig3	2129	3	1	0.078	0.0249	-1.64733	Down	0.347796	0.504862
	CL2157.Contig1	1641	0	1	0	0.0323	5.013462	Up	0.522506	0.665778
	CL2157.Contig4	2030	0	2	0	0.0522	5.705978	Up	0.267068	0.417398
	CL2157.Contig5	1949	0	1	0	0.0272	4.765535	Up	0.522506	0.664098
Claw	CL7013.Contig2	841	801	1395	52.7402	87.8509	0.736154	Up	1.07E-31	5.39E-30
agl5	CL4219.Contig2	790	16	4	1.1215	0.2682	-2.06405	Down	0.005305	0.020402
