# Supplementary material for: Flower transcriptome dynamics during nectary development in pepper (Capsicum annuum L.)
Source: Genet Mol Biol. 2020 May 29;43(2):e20180267. doi: 10.1590/1678-4685-GMB-2018-0267 (PMC7263202; doi:10.1590/1678-4685-GMB-2018-0267)
Supplement: Table S7 - [file 1415-4757-GMB-43-2-e20180267-s14.pdf]

Genetics and Molecular Biology 43, 2, (2020)

Supplementary Material to "Flower transcriptome dynamics during nectary development in pepper (Capsicum annuum L.)"

Table S7 - Nectary-enriched unigenes expression in B3-vs-B1.

Gene family	geneID	Gene Length	B1_raw fragments	B3_raw fragments	B1_FPKM	B3_FPKM	log2 Ratio (B3/B1)	Up-Down-Regulation (B3/B1)	P-value	FDR
beta-fructosidase	CL4573.Contig1	2326	2752	4238	65.5154	97.0481	0.566866	Up	3.46E-59	1.70E-57
	CL4573.Contig2	2155	42	551	1.0792	13.6188	3.657565	Up	3.0515E-110	2.6594E-108
	CL2191.Contig2	1885	48	56	1.4101	1.5824	0.166317	Up	0.561166	0.696974
	CL2191.Contig6	1953	1	13	0.0284	0.3545	3.641823	Up	0.001257	0.005214
	CL2191.Contig1	1847	2	7	0.06	0.2019	1.750607	Up	0.123706	0.239074
	CL2191.Contig5	2039	0	6	0	0.1567	7.291861	Up	0.017877	0.051092
multi - copper oxidase	Unigene21647	1962	8	24483	0.2258	664.6628	11.52336	Up	0	0
	CL3588.Contig1	2108	37	16445	0.9719	415.5267	8.739918	Up	0	0
	CL2403.Contig2	960	1118	2283	64.4875	126.669	0.973972	Up	5.82E-81	3.84E-79
	CL5139.Contig1	1825	261	854	7.9192	24.9247	1.65415	Up	3.20E-69	1.81E-67
	CL1363.Contig2	2093	310	764	8.2016	19.4428	1.245259	Up	3.54E-41	1.27E-39
	Unigene32629	2067	362	599	9.6978	15.4355	0.670523	Up	1.43E-12	1.88E-11
	CL2829.Contig2	1942	520	548	14.8272	15.0303	0.019628	Up	0.824854	0.892751
	Unigene32314	2553	675	714	14.6406	14.8965	0.024999	Up	0.747486	0.839829
	CL7632.Contig1	1759	167	429	5.2572	12.9905	1.30509	Up	1.62E-25	3.86E-24
	CL8488.Contig2	1952	206	410	5.8438	11.1877	0.936935	Up	5.64E-15	8.49E-14
	CL2403.Contig1	292	40	46	7.5855	8.3909	0.145581	Up	0.644568	0.766071
	CL2403.Contig4	1872	123	220	3.6383	6.2597	0.782829	Up	9.28E-07	6.98E-06
	Unigene29898	362	14	31	2.1415	4.5613	1.090823	Up	0.016356	0.047313

Gene family	geneID	Gene Length	B1_raw fragments	B3_raw fragments	B1_FPKM	B3_FPKM	log2 Ratio (B3/B1)	Up-Down-Regulation (B3/B1)	P-value	FDR
	Unigene18601	520	12	32	1.2779	3.2778	1.358953	Up	0.003735	0.013525
	CL1363.Contig1	202	8	12	2.193	3.1642	0.528935	Up	0.434188	0.594132
	CL4586.Contig2	1570	42	54	1.4813	1.832	0.306556	Up	0.30422	0.462769
	CL5139.Contig2	847	10	21	0.6538	1.3206	1.014272	Up	0.064381	0.144733
	Unigene23763	985	80	22	4.4974	1.1897	-1.91849	Down	1.25E-09	1.30E-08
	CL2157.Contig6	2212	20	5	0.5007	0.1204	-2.05611	Down	0.001801	0.007163
	CL2157.Contig2	2048	3	4	0.0811	0.104	0.35881	Up	0.769438	0.852129
	CL2157.Contig3	2129	3	1	0.078	0.025	-1.64155	Down	0.351198	0.513041
claw	CL7013.Contig1	808	1541	1813	105.6079	119.5149	0.178473	Up	0.000351	0.001686
	CL7013.Contig2	841	801	1749	52.7402	110.7719	1.070617	Up	1.18E-72	7.00E-71
agl5	CL4219.Contig2	790	16	1	1.1215	0.0674	-4.05654	Down	0.000106	0.000569
