# Supplementary material for: Flower transcriptome dynamics during nectary development in pepper (Capsicum annuum L.)
Source: Genet Mol Biol. 2020 May 29;43(2):e20180267. doi: 10.1590/1678-4685-GMB-2018-0267 (PMC7263202; doi:10.1590/1678-4685-GMB-2018-0267)
Supplement: Table S8 - [file 1415-4757-GMB-43-2-e20180267-s15.pdf]

Genetics and Molecular Biology 43, 2, (2020)

Supplementary Material to "Flower transcriptome dynamics during nectary development in pepper (Capsicum annuum L.)"

Table S8 - Nectary-enriched unigenes expression in B3-vs-B2.

Gene family	geneID	Gene Length	B2_raw fragments	B3_raw fragments	B2_FPKM	B3_FPKM	log2 Ratio (B3/B2)	Up-Down-Regulation (B3/B2)	P-value	FDR
beta-fructosidase	CL4573.Contig1	2326	2979	4238	67.8311	97.0481	0.516753	Up	1.68E-51	6.20E-50
	CL4573.Contig2	2155	62	551	1.5237	13.6188	3.159949	Up	7.91E-100	5.77E-98
	CL2191.Contig6	1953	1	13	0.0271	0.3545	3.709421	Up	0.000941	0.00267
	CL2191.Contig1	1847	5	7	0.1434	0.2019	0.493596	Up	0.573934	0.663116
	CL2191.Contig5	2039	3	6	0.0779	0.1567	1.00831	Up	0.33911	0.443282
multi - copper oxidase	Unigene21647	1962	73	24483	1.9706	664.6628	8.397844	Up	0	0
	CL3588.Contig1	2108	114	16445	2.8642	415.5267	7.180665	Up	0	0
	CL2403.Contig2	960	699	2283	38.5633	126.669	1.715763	Up	1.26E-196	1.92E-194
	Unigene11619	2211	2393	2639	57.3221	63.575	0.149368	Up	0.000243	0.000783
	Unigene11619	2211	2393	2639	57.3221	63.575	0.149368	Up	0.000243	0.000783
	CL5139.Contig1	1825	401	854	11.6372	24.9247	1.098832	Up	8.67E-39	2.42E-37
	Unigene11984	1239	259	518	11.0712	22.2687	1.008206	Up	3.31E-21	4.99E-20
	CL1363.Contig2	2093	358	764	9.059	19.4428	1.101812	Up	5.05E-35	1.26E-33
	Unigene32629	2067	396	599	10.1467	15.4355	0.605242	Up	6.00E-11	4.73E-10
	CL2829.Contig2	1942	339	548	9.2452	15.0303	0.701097	Up	1.00E-12	9.13E-12
	Unigene32314	2553	614	714	12.7375	14.8965	0.225891	Up	0.004403	0.010578
	CL7632.Contig1	1759	156	429	4.6971	12.9905	1.467615	Up	6.19E-31	1.36E-29
	CL8488.Contig2	1952	194	410	5.2637	11.1877	1.087764	Up	3.64E-19	4.96E-18
	CL2403.Contig1	292	21	46	3.8089	8.3909	1.139451	Up	0.002022	0.005303

Gene family	geneID	Gene Length	B2_raw fragments	B3_raw fragments	B2_FPKM	B3_FPKM	log2 Ratio (B3/B2)	Up-Down-Regulation (B3/B2)	P-value	FDR
	Unigene32128	2061	176	280	4.5228	7.2363	0.678036	Up	7.58E-07	3.78E-06
	CL2403.Contig4	1872	116	220	3.2819	6.2597	0.931562	Up	8.31E-09	5.29E-08
	Unigene29898	362	13	31	1.902	4.5613	1.261928	Up	0.006241	0.014567
	Unigene18601	520	4	32	0.4074	3.2778	3.00821	Up	9.98E-07	4.89E-06
	CL1363.Contig1	202	6	12	1.5731	3.1642	1.00823	Up	0.163279	0.24506
	CL4586.Contig2	1570	30	54	1.012	1.832	0.85621	Up	0.008183	0.018374
	Unigene16391	647	9	20	0.7367	1.6465	1.160253	Up	0.041209	0.075614
	CL4586.Contig2	1570	30	54	1.012	1.832	0.85621	Up	0.008183	0.018374
	CL5139.Contig2	847	8	21	0.5002	1.3206	1.400617	Up	0.015456	0.032347
	CL2157.Contig6	2212	4	5	0.0958	0.1204	0.329738	Up	0.746926	0.808106
	CL2157.Contig3	2129	1	1	0.0249	0.025	0.005782	Up	0.995738	0.99656
	CL2157.Contig1	1641	1	0	0.0323	0	-5.01346	Down	0.502844	0.599401
	CL2157.Contig4	2030	2	0	0.0522	0	-5.70598	Down	0.252136	0.347564
	CL2157.Contig5	1949	1	0	0.0272	0	-4.76553	Down	0.502844	0.598337
claw	CL7013.Contig1	808	1513	1813	99.1735	119.5149	0.269164	Up	7.95E-08	4.52E-07
	CL7013.Contig2	841	1395	1749	87.8509	110.7719	0.334463	Up	9.29E-11	7.20E-10
